# Therapeutic outcomes of acute promyelocytic leukemia in children

**DOI:** 10.1016/j.lrr.2026.100569

**Published:** 2026-02-15

**Authors:** Sirine Chatti, Emna Azza, Marwa Bahri, Roua Hsasna, Yosr Ben Abdennebi, Lamia Aissaoui

**Affiliations:** Hôpital Aziza Othmana Tunis, Tunis, Tunisia

**Keywords:** Leukemia, Acute, Children, Promyelocytic, ATRA

## Abstract

**Introduction:**

In children, acute promyelocytic leukemia is a distinct and underreported entity. The objective of our study is to report the therapeutic outcomes of APL in the pediatric population.

**Methods:**

This was a descriptive, retrospective, single-center study including 30 patients diagnosed with APL over an 18-year period. Patients were treated in the Hematology Department of Aziza Othmana Hospital in Tunis.

**Results:**

The median age at diagnosis was 11 years, with a sex ratio of 0.87. Half of the patients were classified as high-risk. Post-induction cytologic remission was achieved in 86.6 % of patients. There were five cases of relapse, 60 % of which were early relapses. The 5-year overall survival rate was 70.6 %.The 5-year event-free survival rate was 72 %. The 5-year relapse-free survival rate was 80 %.

**Conclusion:**

Although our results were satisfactory, they revealed a poor prognosis for relapsed patients. A larger study focusing on relapse characteristics is needed.

## 1 Introduction

Acute promyelocytic leukemia (APL) represents a distinct subtype of acute myeloid leukemia (AML) due to its specific cytogenetic and molecular characteristics [1].

Despite its acute onset and the high risk of hemorrhage-related mortality, international study groups have contributed to the development of multiple treatment options, making APL a highly curable disease. This has paved the way for an era of targeted therapy, which has revolutionized the prognosis of this leukemia, particularly with the introduction of all-trans retinoic acid (ATRA) and arsenic trioxide (Arsenic Trioxide) over the past 30 years [2].

In children, APL is a rare clinical entity [2], and remains underreported, especially in low- and middle-income countries. Given the epidemiological and clinico-biological particularities of pediatric APL and its risk of relapse, a more detailed evaluation of therapeutic response specifically in this age group is necessary.

Thus, the objective of our study is to report the therapeutic outcomes of APL in children aged 2 to 18 years, treated with 2 successive multicenter protocols: LPA99 and LPA05.

## 2 Patients and methods

### 2.1 Patients

This is a descriptive, retrospective, single-center study including children and adolescents diagnosed with APL who were followed up and treated in the clinical hematology department of Aziza Othmana Hospital (AOH) in Tunis from january 2004 to december 2021, according to the LPA1999 protocol (2004–2013) and the LPA2005 protocol (2014–2021)**.**

#### 2.1.1 Inclusion criteria

All children aged between 2 and 18 years, newly diagnosed with APL, meeting the following diagnostic criteria:

- Bone marrow smear showing a cytological appearance consistent with APL as defined by the FAB (French-American-British) classification [3] (Table 1), with or without a bone marrow immunophenotypic profile suggestive of APL [4] (Table 2).

**Table 1:** FAB Classification of acute myeloid leukemias.

AML 1:	• Myeloblastic without differentiation (15 %)
**AML 2**:	• Myeloblastic with differentiation (25 %)
**AML 3**:	• Promyelocytic (10 %)
**AML 4**:	• Myelomonocytic (20 %)
**AML 4Eo**:	• Myelomonocytic with eosinophilia (5 %)
**AML 5**:	• Monoblastic
o**M5a**:	o Without differentiation
o**M5b**:	o With differentiation (10 %)
• **AML 6**:	• Erythroblastic or erythroleukemia (5 %)
• **AML 7**:	• Megakaryoblastic (5 %)

**Table 2:** Immunophenotypic characteristics of APL.

• Classic Form (M3):
o Expression of myeloid lineage precursors: CD117, CD33, CD13
o High side scatter (SSC)
o No expression of immaturity markers (CD34 and HLA-DR) or B and T lymphoid markers
• **Variant Form (M3v)**
o Expression of myeloid lineage precursors: CD117, CD33, CD13
o Low side scatter (SSC)
o Possible expression of immaturity markers (CD34 and HLA-DR) and B and T lymphoid markers

Cytogenetic confirmation by identification of the t (15; 17) translocation on the oncological karyotype and/or the presence of the PML/RARα transcript by molecular biology.

These patients are treated according to PETHEMA protocols: LPA99 (2004–2013) and LPA05 (2014–2021).

### 2.2 Treatment protocols

The patients in our study were classified according to the relapse risk score developed by Sanz et al. [5].Table 3

**Table 3:** Definition of relapse risk by the Sanz score.

Relapse Risk	Low	Intermediate	High
WBC (G/L)	<10	<10	≥10
Platelet count (G/L)	>40	≤40	-

#### 2.2.1 PETHEMA-LPA99 protocol

In the induction regimen (course I), all patients received ATRA (25 mg/m₂/d) orally until complete remission (CR) combined with chemotherapy (CT) including 12 mg/m₂/d idarubicin (IDA) on days 2, 4, 6 and 8 of ATRA treatment.

Patients with CR received 3 consolidation courses, including 15 days of ATRA and doses of anthracyclines. Patients classified as low risk received IDA on Days 1, 2, 3, and 4 at a dose of 5 mg/m² and ATRA at a dose of 25 mg/m².

Patients classified as intermediate or high risk received a higher dose of anthracycline: 7 mg/m².

Maintenance treatment was initiated after the third consolidation course and upon confirmation of molecular remission.

During two years, patients received the following oral medications:

methotrexate (Methotrexate): 15 mg/m²/week (started one month after hematologic recovery).
6-Mercaptopurine (6-MP): 50 mg/m²/day (started one month after hematologic recovery).
ATRA: 25 mg/m²/day for 15 days every 3 months (started three months after the completion of consolidation therapy). 6-MP and MTX were temporarily discontinued during ATRA administration.

#### 2.2.2 PETHEMA-LPA05 protocol

Induction regimen was identical to that of the LPA99 protocol.

Then, once hematologic recovery was achieved, patients who attained CR received three successive consolidation courses, stratified according to the Sanz risk score:

Low-risk and intermediate-risk patients received consolidation therapy based on the same combination of ATRA and anthracyclines as LPA99 protocol.
High-risk patients additionally received cytarabine (Cytarabine) during the first and third consolidation cycles on Days 1, 2, 3, and 4.

The maintenance therapy was identical to that of the LPA99 protocol.

### 2.3 Supportive therapy

Coagulopathy was treated with fresh-frozen plasma with the goal to maintain fibrinogen>1 g/l. Platelet transfusions were given to maintain a platelet count of >50 G/l until resolution of significant coagulopathy.
The transfusion of phenotyped red blood cell concentrates (RBCs) was indicated for hemoglobin levels below 8 g/dL in the absence of signs of poor anemia tolerance. In cases of poorly tolerated anemia, an emergency transfusion was performed regardless of the hemoglobin level.
A broad-spectrum antibiotic therapy was based on the suspected site of infection.

### 2.4 Statistical analysis

All statistical analyses were performed with IBM© SPSS© Statistics version 25. Numerical variables were expressed as median and range, while qualitative variables were expressed as frequency and percentage. The chi-square test or Fisher’s exact test was used to analyze the relation between qualitative variables, while quantitative data were compared using Student's *t*-test. Relapse-Free Survival (RFS) was defined as the time from the date of achieving complete remission (CR) until the date of relapse. Event-free survival (EFS) was defined as the duration from the diagnosis date to the occurrence of the first event (relapse or death) or the last contact. Overall survival (OS) was defined as the time from the diagnosis date to death or last contact. The Kaplan-Meier method was employed to estimate RFS, EFS and OS. The impact of prognostic factors on OS, EFS, and RFS was analyzed using the Log-Rank method for univariate analysis and the Cox model for multivariate analysis. A p-value < 0.05 was considered significant.

## 3 Results

### 3.1 Patients

We collected data on 30 patients treated for APL in the clinical hematology department of AOH Tunis over an 18-year period. They accounted for 22.7 % of children AML diagnosed during this period. The median age of the patients at diagnosis was 11 years [range: 2–18]. Among them were 14 males and 16 females (gender ratio: 0.87).

The hemorrhagic and anemic syndromes were the most common initial presentations of the disease (43.3 %). At initial clinical examination, 11.5 % of patients were obese. Hemorrhagic syndrome was the most frequent clinical sign, observed in 46.7 % of patients, while disseminated intravascular coagulation (DIC) occurred in 40 % of the cases.

### 3.2 Laboratory and diagnostic findings

The median white blood cell (WBC) count was 12 G/L [range: 0.66–146.89]. Fifteen patients were classified as high risk of relapse (50 %), thirteen patients (43.3 %) as intermediate risk, and two patients (6.7 %) as low risk. Twenty patients (66.7 %) had a variant cytological form.

Immunophenotyping of blasts was performed in 20 patients, while the remaining 10 patients were difficult to sample.The markers cMPO, CD34, and HLA-DR were identified in all patients (100 %). The markers CD13 and CD33 were tested and found in 16 patients (100 %). The marker CD117 was tested in 11 patients and identified in 10 (90.9 %).The side scatter (SSC) was tested and found in 8 patients. It was intense in five patients, and low in three.

The t (15; 17) translocation was present in 21 patients. Additional chromosomal abnormalities (ACA) were observed in 20.8 % of interpretable karyotypes. The PML-RARα transcript was present in 29 patients (96.6 %).

### 3.3 Treatment outcome

All patients were treated according to PETHEMA protocols: the LPA99 protocol was used in 18 patients (60 %) between 2010 and 2014, and the LPA05 protocol was used in 12 patients (40 %) between 2014 and 2021.

The early mortality rate was 10 %, attributed to a severe hemorrhagic complication related to disseminated intravascular coagulation (DIC).

Differentiation syndrome (DS) occurred in 10 % of patients during the induction therapy. The diagnosis was confirmed according to the FRANKEL creteria [6].Table 4

**Table 4:** Characteristics of patients with differentiation syndrome.

Patient	Obesity	Tumor Syndrome	DIC	WBC ≥ 10 G/L	Blood Blasts	Variant Form
Patient 1	Yes	No	No	Yes	Yes	Yes
Patient 2	No	Yes	Yes	Yes	Yes	No
Patient 3	Yes	No	Yes	Yes	Yes	Yes

The occurrence of a pseudotumor cerebri was noted in one patient after six days of ATRA treatment initiation.

One patient (3.3 %) developed Sweet's syndrome, which was histologically confirmed by a skin biopsy.

Anthracycline-induced cardiac toxicity was observed in two patients, with a median onset of 30 days after the initiation of anthracycline treatment [range: 29–30 days].

The post-induction cytological remission rate was 86.6 %. The absence of molecular remission after the first consolidation was observed in six patients.

### 3.4 Survival rates

**Fig. 1:**
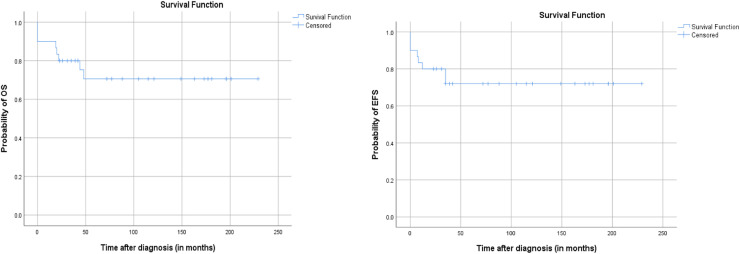
The 5-year probability of OS, RFS and EFS of the entire group.

**Fig. 2:**
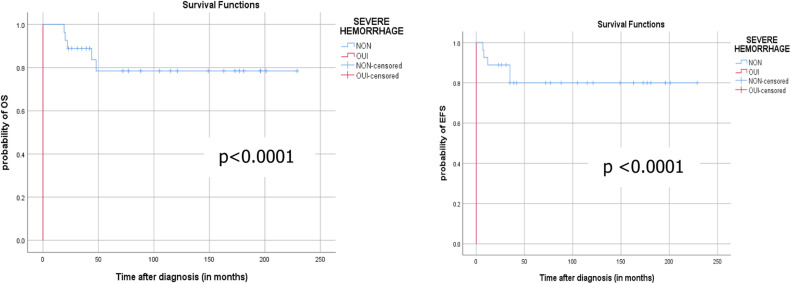
The 5-year probability of OS and EFS according to the occurrence of severe hemorrhage.

**Fig. 3:**
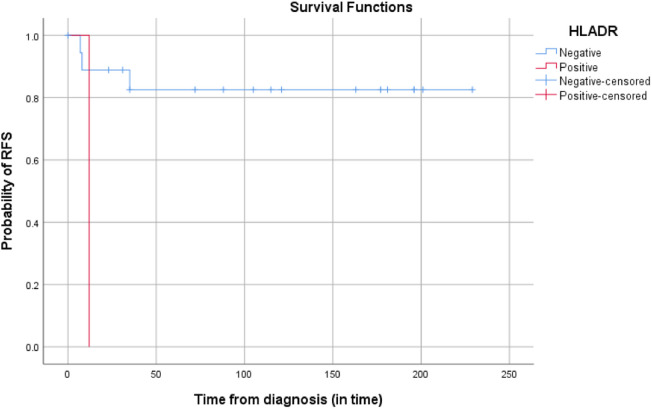
The 5-year probability of RFS according to the expression of HLA-DR.

Overall survival (OS) at 5 years was 70.6 %. Event-free survival (EFS) was 72 %. Relapse-free survival (RFS) was estimated at 80 %.Fig. 1, Fig. 2, Fig. 3

Prognostic factors that affected OS in univariate analysis included female sex, occurrence of severe hemorrhagic syndrome, expression of HLA-DR, cessation of ATRA, and occurrence of relapse. Severe hemorrhagic syndrome was the only statistically significant prognostic factor in multivariate analysis. Prognostic factors that influenced EFS identified in univariate analysis included: female sex, severe hemorrhagic syndrome, differentiation syndrome, and cessation of ATRA. Multivariate analysis did not identify any significant prognostic factors.

In univariate analysis, HLA-DR expression was identified as a predictive factor for relapse. No factors were significant in multivariate analysis.Table 5

**Table 5:** Summarizes the OS and EFS rates according to age, sex, initial WBC count, initial DIC, cytologic type, cytogenetic abnormalities, occurrence of DS, and ATRA iterruption. No significant differences were detected in survival rates.

	5-year EFS	p-value	5-year OS	p-value
Age				
≤ 12 ans	78.6 %	0.28	77.2 %	0.34
> 12 ans	58.2 %		58 %	
Sex				
Male	91.7 %	0.02	88.9 %	0.03
Female	54.7 %		54.7 %	
Initial WBC count				
< 10 G/L	84 %	0.12	83 %	0.10
≥ 10 G/L	60 %		58.3 %	
Initial DIC				
Yes	72.2 %	0.96	71.3 %	0.96
No	69.4 %		66.7 %	
Cytologic type				
Classical	85.7 %	0.16	85.7 %	0.13
Microgranular	64.6 %		63 %	
Protocol				
LPA99	70.6 %	0.84	70.1 %	0.93
LPA05	72.5 %		67.7 %	
Occurrence of DS				
Yes	33.3 %	0.051	33.3 %	0.08
No	76.2 %		74.5 %	
ATRA interruption				
Yes	63.3 %	0.04	63.6 %	0.047
No	92.3 %		90 %	

*Abbreviations*: OS, overall survival; EFS, event-free survival; WBC, WBC, White blood cell count; DS: Differentiation syndrome; DIC: Disseminated intravascular coagulation; ATRA: All-Transretinoic acid.

## 4 Discussion

Because of its rarity, reports of pediatric APL are limited, especially in countries with financial and technical constraints. This is the second study in Tunisia including children diagnosed with APL and treated with ATRA and chemotherapy. The actual incidence of APL in children remains undetermined and is marked by significant variability in frequency and occurrence across different ethnic and geographic populations [7]. This study showed that 22.7 % of AML cases in children were diagnosed with APL.

In our study, the analysis of the clinical features at presentation revealed a predominance of female sex as described in several series [8] . Hemorragic magnifestations were the main mode of presentation, as reported in most studies on APL [9].

A meta-analysis conducted in 2017 by Li and al [10] showed that overweight or obese patients had a high prevalence of APL. In our study, three patients were overweight, and three patients were obese (11.5 %). A higher incidence was reported by pediatric series of the “Cancer and leukemia Group” (34.5 %), and the “Children Oncology Group” (33.66 %) [11].

In another hand, the analysis of the biological features at presentation showed a close agreement with the literature. Studies on pediatric APL have reported a relatively high proportion of children presenting with hyperleukocytosis at diagnosis (>10 G/L), ranging from 35 % to 48 % [12]. In our study, the median range of leucocytes was 12 G/L and 50 % of our patients were classified as high-risk based on leucocytes count (>10 G/L).

A biological disseminated intravascular coagulopathy (DIC) was present in 60 % of our patients. These findings closely align with previously published data by a recent Taiwanese study conducted by Yeh and al. It revealed a proportion of 77.6 % of LAP pediatric patients presenting with DIC [13].

The frequency of the variant cytologic form was 32 % in the pediatric series studied by Guglielmi et al. [4]. This form was predominant in our series (66.7 %). Patients with the variant form had a higher WBC count compared to those with the classic form, both in our study (46.6 %) and in the literature [14].

Immunophenotyping analysis showed a rare expression of the HLA-DR marker (only one patient in our study) and a low expression of the CD34 marker (10 %) as reported in the literature. Expression of the T-cell marker CD2 was also observed both in our study (6.6 %) and in the study by Guglielmi and al., wich included 68 children diagnosed with APL.

The analysis of the cytogenetic features showed 20.8 % of patients with additional cytogenetic abnormalities to t(15;17). This finding is similar to data published by PETHEMA clinical trials “LPA96” and “LPA99” where 28 % of patients with APL had additional cytogenetic abnormalities [15].

The major therapeutic protocols for pediatric acute promyelocytic leukemia (APL), combining all-trans retinoic acid (ATRA) with idarubicin during induction (AIDA) followed by three consolidation cycles, were developed by the Italian GIMEMA group and the Spanish PETHEMA group. These protocols, notably LPA1999 and LPA2005, have demonstrated improved therapeutic efficacy compared to the earlier LPA1996 protocol, as reported by Sanz and al [16].

In this study 18 patients were treated by LPA1999 protocol (60 %) and 12 patients by LPA2005 protocol (40 %).

We observed 3 early deaths (10 %) caused by severe haemorrhage. The hemorrhagic syndromes included hemoperitoneum, alveolar hemorrhage, and profuse hematuria with a median time to death of 4 days of treatment [1–4 days]. ​These findings are consistent with the literature. In previous studies on APL, hemorrhagic deaths frequently occurred during the first week of induction therapy, primarily due to cerebral and pulmonary hemorrhages, with respective incidences of 65 % and 32 % [17]. In the pediatric series published by Testi and al, the mortality rate was 3.6 %, with 75 % of early deaths attributed to cerebral hemorrhage occurring within a median of 7 days [18].

Three patients developed differentiation syndrome (DS) in a median time of 3 days of ATRA treatment [[2], [3], [4]]. In reports on children APL, the frequency, diagnosis, and management of this complication have not yet been fully established. In the AAML1331 and AAML0631 studies by the "COG," 41 out of 154 children with APL (26.6 %) developed SD [31]. In contrast, the study published by "GIMMEMA and AIEOP" reported only 2 cases of SD among 107 pediatric patients [19].

According to the literature, Pseudotumor Cerebri is another ATRA complication more common in children and adolescents [12], but its incidence has decreased with the use of a reduced dose of ATRA in PETHEMA protocols [20]. In our study, it occurred only in 1 patient.

As reported in many series of patients with APL, the response to induction treatment was satisfying. Our results showed complete remission in 86.6 % of our patients after induction. The molecular remission rate after the first consolidation was 77.8 %. Six patients remained positive for the PML-RARα transcript (22.2 %). Among the 26 patients who were in CR post-induction, 5 patients relapsed within a median time of 11 months [5–34].

Unfortantly, OS ans EFS rates were lower than data published by the largest pediatric trials, as reported, rescpectively by PETHEMA, GIMEMA and COG groups (Table 6).

**Table 6:** CR, OS and EFS rates reported by pediatric studies compared to our study.

Study Group	Period of time	Number of patients	Induction Type	CR %	5-year OS %	5-year EFS %
GIMEMA [19]	1993–2000	128	ATRA+IDA	96	89	76
PETHEMA [21]	1999–2005	66	ATRA+IDA	92	87	82
COG [22]	2015–2019	154	ATRA+ATO	100	100(RS) 99(HR)	98 (RS) 96 (HR)
Our study	2004–2021	30	ATRA+IDA	86.6	70.6	72

This finding is likely attributable to the limited use of arsenic trioxide (Arsenic Trioxide) as first- or second-line therapy, in contrast to its frequent incorporation into treatment protocols for comparable patient populations in previously reported studies. Notably, the AAML1331 Children’s Oncology Group trial demonstrated that the combination of all-trans retinoic acid (ATRA) and ATO in first- line treatment, is both safe and highly effective in pediatric patients with standard-risk acute promyelocytic leukemia, with a reported 2-year event-free survival rate of 98 % [22].

The prognostic factors that influenced OS in univariate analysis were : female sex (*p* = 0.029), the occurrence of a severe hemorrhagic syndrome (*p* < 0.0001), the expression of HLA-DR (*p* = 0.03), the discontinuation of ATRA (*p* = 0.047), and the occurrence of a relapse (*p* < 0.001). Multivariate analysis showed a statistically significant impact of the occurrence of a severe hemorrhagic syndrome.

The prognostic factors that influenced the EFS in univariate analysis were : female sex (*p* = 0.019), the occurrence of a severe hemorrhagic syndrome (*p* < 0.0001), and the discontinuation of ATRA (*p* = 0.040). In multivariate analysis, the factors impacting EFS were not statistically significant.

Even in all the published studies regarding APL, severe hemorhage remains of significant negative influence on survival. However, there is no estimation of long term survival in pediatric patients treated for APL because of the high early mortality rate [23].

The prognostic implication of age, sex, high initial WBC counts, DS and ATRA interruption need to be further proven in prospective, multicenter analyses.

## 5 Conclusion

This study characterizing pediatric APL treated with ATRA and anthracyclines represents a significant contribution to the literature, especially considering the lack of sufficient studies on pediatric APL in developing countries. It provides useful epidemiological and descriptive data on APL in a North African pediatric population.

However, this investigation has several limitations that should be acknowledged. First, the relatively small sample size (*n* = 30) collected over an 18-year period reduces statistical power and contributes to substantial variability in outcome estimates. Additionally, the retrospective, single-center design introduces inherent limitations, including potential selection bias, incomplete or missing data, and heterogeneity in treatment approaches and follow-up duration across the study cohort.

Our results could be optimized by adopting a prospective, multicenter design with a larger sample size to improve statistical power and generalizability. Standardizing treatment protocols and follow-up would reduce variability and missing data.

## CRediT authorship contribution statement

**Sirine Chatti:** Writing – original draft. **Emna Azza:** Writing – original draft. **Marwa Bahri:** Project administration. **Roua Hsasna:** Project administration. **Yosr Ben Abdennebi:** Supervision. **Lamia Aissaoui:** Validation.

## Declaration of competing interest

The authors declare that they have no known competing financial interests or personal relationships that could have appeared to influence the work reported in this paper.
